# Differences in the intestinal microbiota between uninfected piglets and piglets infected with porcine epidemic diarrhea virus

**DOI:** 10.1371/journal.pone.0192992

**Published:** 2018-02-15

**Authors:** Mei-Zhou Huang, Sheng-Yi Wang, Hui Wang, Dong-An Cui, Ya-Jun Yang, Xi-Wang Liu, Xiao-Jun Kong, Jian-Yong Li

**Affiliations:** 1 Lanzhou Institute of Husbandry and Pharmaceutical Sciences of CAAS, Lanzhou, China; 2 Key Lab of New Animal Drug Project of Gansu Province, Lanzhou, China; 3 Key Lab of Veterinary Pharmaceutical Development of Ministry of Agriculture, Lanzhou, China; National Centre For Cell Science, INDIA

## Abstract

Porcine epidemic diarrhea, a disastrous gastrointestinal disease, causes great financial losses due to its high infectivity, morbidity and mortality in suckling piglets despite the development and application of various vaccines. In this study, high-throughput sequencing was used to explore differences in the intestinal microbiota between uninfected piglets and piglets infected with porcine epidemic diarrhea virus (PEDV). The results revealed that the small intestinal microbiota of suckling piglets infected with PEDV showed low diversity and was dominated by Proteobacteria (49.1%). Additionally, the composition of the small intestinal microbiota of sucking piglets infected with PEDV showed marked differences from that of the uninfected piglets. Some of the taxa showing differences in abundance between uninfected piglets and piglets infected with PEDV were associated with cellular transport and catabolism, energy metabolism, the biosynthesis of other secondary metabolites, and amino acid metabolism as determined through the prediction of microbial function based on the bacterial 16S rRNA gene. Therefore, adjusting the intestinal microbiota might be a promising method for the prevention or treatment of PEDV.

## Introduction

Porcine epidemic diarrhea (PED), a disastrous gastrointestinal disease, causes great financial losses due to its high infectivity, morbidity and mortality in suckling piglets [1–3]. The major clinical symptoms of PED are severe enteritis, vomiting and watery diarrhea [4]. Porcine epidemic diarrhea virus (PEDV) rapidly spreads among piglets through the fecal-oral route [5]. Although various vaccines have been developed and applied, PED outbreaks still occur in some immunized swine herds [6,7]. Therefore, the identification of new preventive measures as well as further research and development of new vaccines are necessary.

The intestinal microbiota is an important barrier against invaders entering via the gastrointestinal route [8]. Many studies have shown that commensal microbiota can prevent pathogenic invasion by competing for receptors and enteric nutrients, stimulating the innate immune system to inhibit pathogens, producing antimicrobial compounds such as bacteriocins, and creating a microenvironment that is adverse to enteric pathogens [9–11]. Due to increasing knowledge regarding the composition and function of the intestinal microbiota and commensal host-microbial relationships, the potential applications of commensal bacteria, such as anti-inflammatory therapy, antitumor therapy and antivirus therapy, have attracted significant attention [12–14].

However, few studies have investigated the differences in the intestinal microbiota between uninfected piglets and piglets infected with a virus. With the advent of high-throughput sequencing, the analysis of the 16S rRNA gene constitutes a rapid and effective method for assessing bacterial diversity and abundance [15]. Therefore, to investigate differences in the intestinal microbiota between PEDV-infected and uninfected piglets, the diversity and abundance of the intestinal microbiota of infected and uninfected piglets were assessed. The results suggest that changes in the intestinal microbiota occur following the onset of PEDV.

## Materials and methods

### Animals and specimen collection

This trial was conducted at a swine farm of Gansu Chen Yu Ecological Agriculture Development Co., Ltd. (Pingliang, China), using three-way crossbred sows and piglets (Duroc-Landrace-Yorkshire). The saliva of sows and piglets was collected in five farrowing houses of the commercial swine farm from the third to the eighth day after the piglets were born. In brief, cotton threads were hung above each sow stall and adjusted to the appropriate height and position to ensure that the sows and piglets could touch different cotton threads. Each day, the saliva on the cotton threads was squeezed into collecting pipes, followed by replacement of the cotton threads with new ones. The presence of pseudorabies virus (PRV), classical swine fever virus (CSFV), porcine reproductive and respiratory syndrome virus (PRRSV) and PEDV in the saliva samples was detected using PRV, CSFV and PRRSV real-time fluorescent RT-PCR kits and a PEDV-transmissible gastroenteritis virus (TGEV)-porcine rotavirus triple real-time fluorescent RT-PCR kit, respectively. Based on the results from the saliva samples and clinical examination by an experienced veterinarian conducted in four sow stalls of the same farrowing house, 16 subjects were enrolled in the experiment (with one sow and three suckling piglets placed in each sow stall). All of the suckling piglets were of the same age (seven days) and breed. Two sow stalls were used to house the infected group: All of the suckling piglets in these stalls showed early symptoms of PEDV and tested positive for PEDV, and the sows were negative for other contagious diseases. The other two stalls housed the control group: All of the subjects were found negative for PEDV and were in good mental and physical states. All of the suckling piglets were slaughtered at seven days of age, and the contents of their small intestines from the duodenum to the ileum were collected under aseptic conditions and then snap-frozen in liquid nitrogen. Milk was collected from all of the sows and stored at -20°C until analysis. The protocols used in this study comply with the Guidelines for the Care and Use of Laboratory Animals as described by the US National Institutes of Health as well as the protocols including all sampling methods and experimental manipulations used in this study were reviewed and approved by Gansu Chen Yu Ecological Agriculture Development co., LTD and Institutional Animal Care and Use Committee of Lanzhou Institute of Husbandry and Pharmaceutical Science of CAAS (Animal Use Permit: SCXK201548–0710). All of the suckling piglets were sacrificed by euthanasia.

### Analysis of milk nutrients

The milk samples were thawed and heated to 37°C in a water bath. The milk components, including protein, fat, total solids (TS), lactose and acidity, were then analyzed using a MilkoScan FT120 instrument (Foss, Denmark).

### 16S rRNA gene sequencing of the small intestine microbiota

The microbial genomic DNA extracted from the samples of the small intestine was qualified and quantified, and the V4 hypervariable region of the 16S rRNA gene was then amplified and purified using the PCR primers 515F (5’-GTGCCAGCMGCCGCGGTAA-3’) and 806R (5’-GGACTACHVGGGTWTCTAAT-3’). The sequencing library was quantified by Qubit and qPCR, and the barcoded V4 PCR amplicons were sequenced using an Illumina HiSeq 2500 PE250 platform. All sequencing of the small intestinal microbial 16S rRNA gene was performed by Tianjin Novogene Bioinformatics Technology Co., Ltd. In detail, using a small fragment library constructed using PCR sample preparation kits for the paired-end sequencing of the barcoded V4 PCR amplicons based on the Illumina HiSeq 2500 PE250 platform, the sequences used in the subsequent analysis (effective tags) were obtained by successively splicing raw sequence reads using FLASH (version 1.2.7) software [16], filtering raw tags using QIIME (version 1.7.0) software [17] according to the split_._libraries_-_fastq.py script with the parameters (-q 19-p 0.75), and eliminating chimeric sequences.

### Taxonomy classification and statistical analysis

#### Taxonomy classification

Using UPARSE software (version 7.0.1001) [18], repetitive sequences were removed from the effective tags to acquire representative sequences. The representative sequences were then ranked according to size, and those representative sequences with a size of 1 were eliminated. Representative sequences showing 97% identity were then clustered as operational taxonomic units (OTUs) using UPARSE (version 7.0.1001). The representative sequences of the OTUs were annotated using RDP classifier (version 2.2) and the Greengenes database (version 13.5) [19,20]. To construct the phylogenetic relationships of the representative sequences, all of the representative sequences were aligned using MUSCLE (version 3.8.31) software [21]. The data from all of the samples were then normalized for the analysis of alpha and beta diversity. In brief, the sequence numbers of the sample with the fewest sequences were selected to set the threshold value, and the same number of sequences from each of the samples was randomly extracted for subsequent analysis.

#### Analysis of alpha and beta diversity

Alpha diversity is used to assess the diversity and abundance of microbes. Various alpha diversity indexes, such as observed species, Chao1, Shannon, Simpson and Goods-coverage, were calculated using QIIME (version 1.7.0) software, and rarefaction and rank abundance curves were then drawn with R (version 2.15.3) software. To compare the construction of the microorganisms between the different samples, unweighted and weighted UniFrac distances were calculated using QIIME (version 1.7.0) [22]. In addition, the samples were clustered based on the unweighted or weighted UniFrac distance matrix using the Unweighted Pair-Group Method with Arithmetic Mean (UPGMA) implemented in QIIME (version 1.7.0) [16].

### Microbial function prediction

To explore the potential functional profiles of the bacterial community, the sequences were clustered into OTUs using a closed-reference approach and the Greengenes 13.5 database at 97% similarity using QIIME (version 1.7.0). The resulting OTUs were used for the prediction of microbial function with PICRUST [23] according to the online protocol. Briefly, after the OTUs were normalized by 16S rRNA copy number, the metagenome for each sample was predicted, and the accuracy of the metagenome predictions was assessed. Differences between the control and experimental groups were assessed by using STAMP software [23].

### Statistical analysis

One-way t-tests were performed using SAS 9.2 (SAS Institute Inc., NC, USA) to analyze the differences in milk nutrients between the control and experimental groups. Discriminant analysis was performed with JMP Pro (SAS Institute Inc., NC, USA) to analyze the difference in the small intestinal microbiome between the control and experimental groups using the species numbers as covariates and PEDV infection as the categorical variable.

## Results

### Nutrients in milk

No difference (P > 0.05) in the nutrient contents of sows’ milk was found between the control and infected groups (Table 1).

**Table 1:** Nutrient content of sows’ milk (mean ± SD).

Group	Protein (%)	Fat (%)	Total solids (%)	Lactose (%)	Acidity (°T)
**Control**	7.09 ± 0.19	5.99 ± 0.31	12.37 ± 0.16	2.17 ± 0.04	17.74 ± 2.093
**Infected**	7.35 ± 0.12	5.50 ± 0.45	13.76 ± 0.01	2.57 ± 0.20	18.70 ± 0.47

### Microbiota profiles in the small intestine of suckling piglets

**Fig 1:**
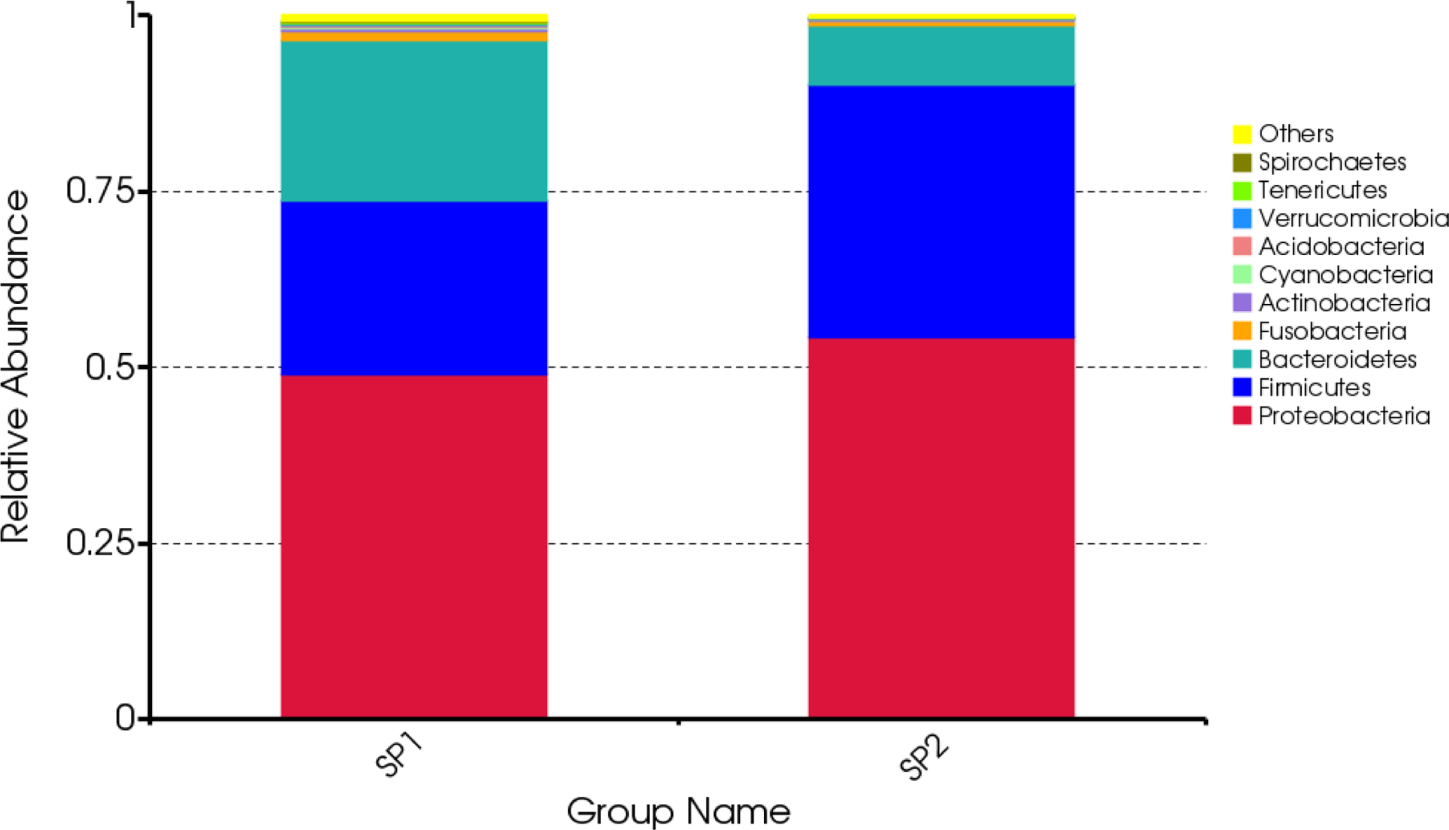
Average relative abundance of small intestinal microbial species at the phylum level. SP1 and SP2 refer to uninfected and infected suckling piglets, respectively.

**Fig 2:**
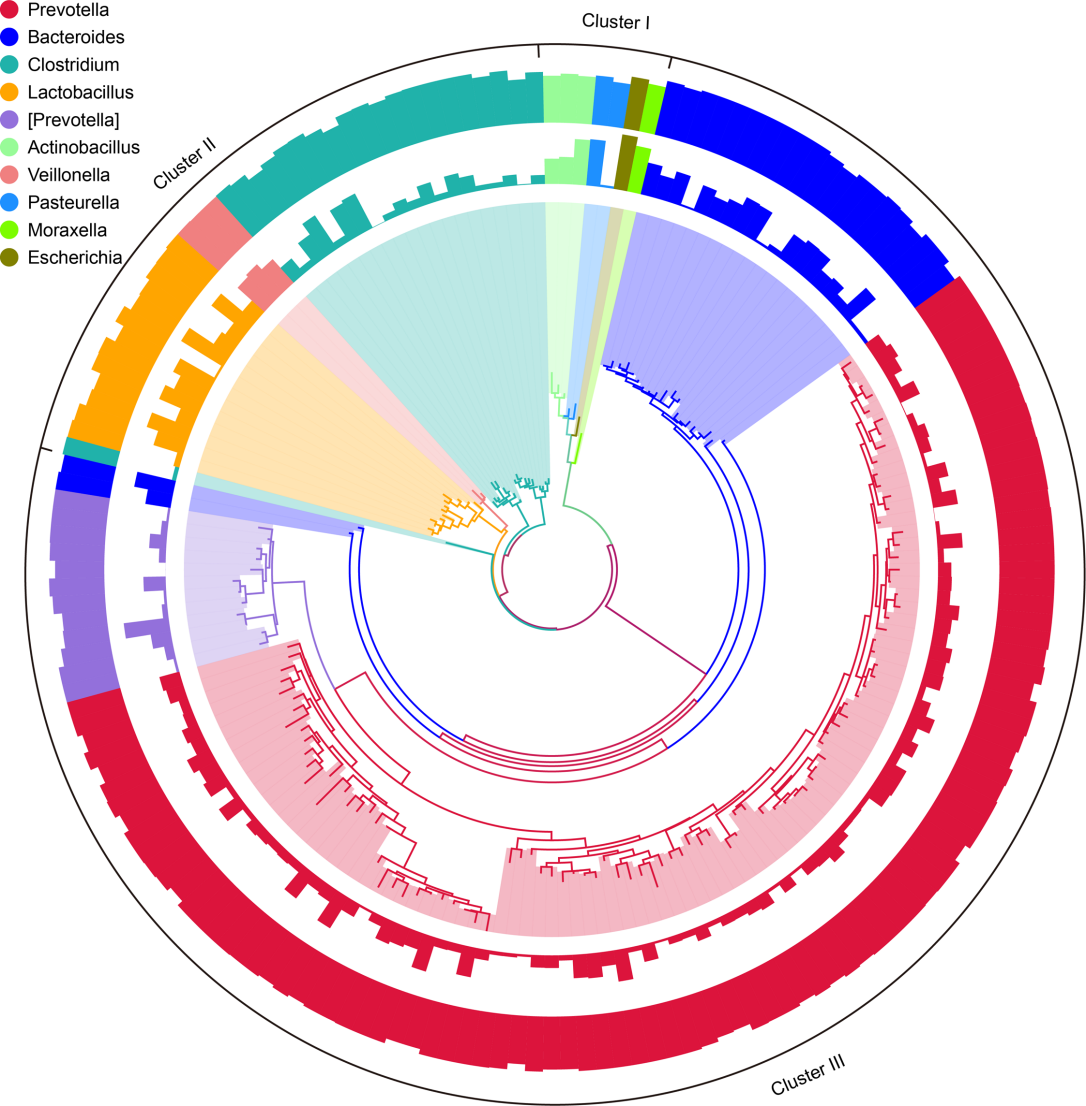
Phylogenetic relationships and species annotation of small intestinal microbial flora. The phylogenetic tree is the first layer constructed based on the representative OTU sequences, and each branch color corresponds to a specific genus. The second layer represents the distribution of the relative abundance of OTUs, with bar height indicating the abundance value. The third layer depicts the distribution of the reliability of the OTU annotation, with bar height indicating the reliability value.

**Fig 3:**
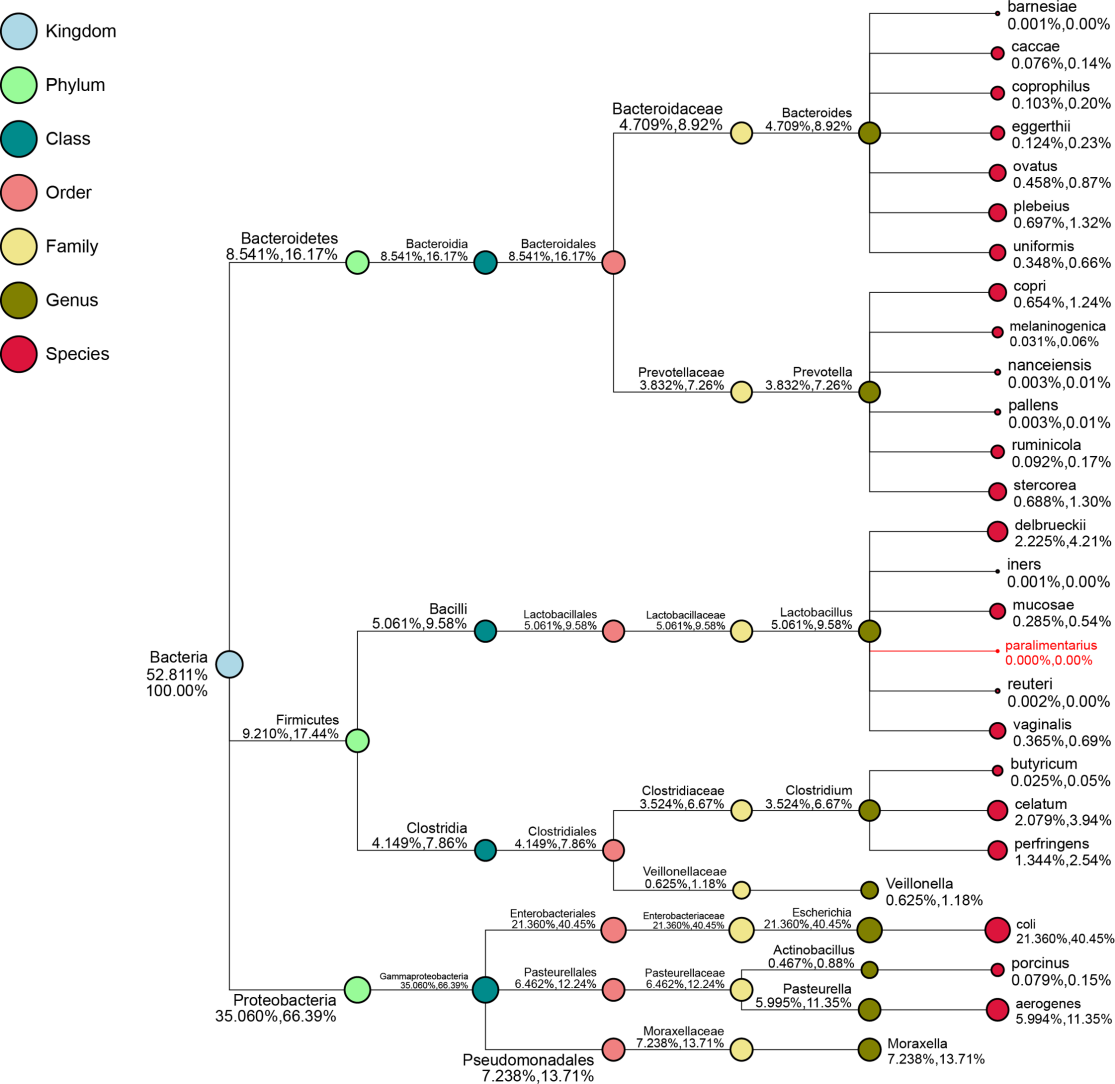
Taxon composition of the small intestinal microbial flora of uninfected suckling piglets. The numbers after the taxonomic ranks are the relative abundances of the corresponding taxa at each taxonomic rank within the total species or selected species.

The taxonomic analysis indicated that the most abundant phylum in the small intestinal microbiota of uninfected suckling piglets was Proteobacteria (49.1%) (Fig 1). The constructed phylogeny revealed that the 10 most abundant genera of microbiota in the control group were grouped into three clusters (Clusters I, II, and III; Fig 2). Clusters I and II each comprised four genera, whereas Cluster III comprised only *Prevotella* and *Bacteroides*. To obtain a taxon composition profile of the small intestinal microbiota, a species classification tree was constructed based on the 10 genera with the highest relative abundances (Fig 3), which accounted for 52.8% of the small intestinal microbiota. At the genus level, *Escherichia* (21.4%) was dominant, whereas the other nine genera had relatively low abundances, ranging from 0.457% to 7.24% of the identified genera.

### Shifts in the abundance and composition of the small intestinal microbiota due to PEDV infection

**Fig 4:**
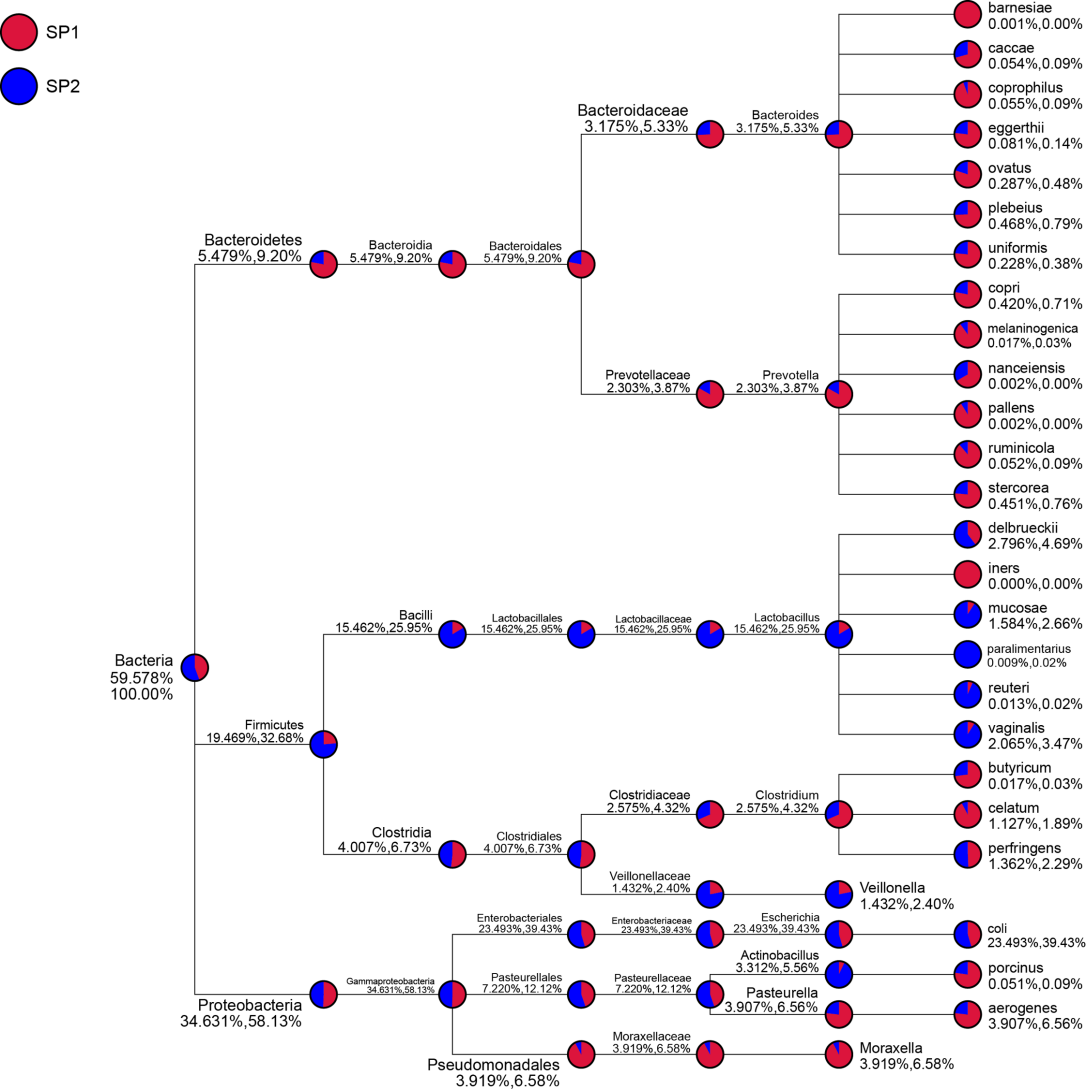
Taxon composition of small intestinal microbial flora of uninfected suckling piglets (SP1) and infected suckling piglets (SP2). The area of the sector represents the relative abundance of the sample in the taxonomic ranks. The numbers after the taxonomic ranks are the relative abundances of the corresponding taxa within the total species or selected species.

The abundance of Firmicutes (35.96%) was higher in infected suckling piglets than in uninfected ones (24.66%), whereas the abundance of Bacteroidetes (8.37%) was lower in infected piglets than in uninfected ones (22.78%; Fig 1). The taxon composition profile of infected sucking piglets was significantly different from that of uninfected suckling piglets (Fig 4). At the genus level, the relative abundances of *Bacteroides*, *Prevotella*, *Pasteurella* and *Moraxella* in infected suckling piglets were lower than those in uninfected suckling piglets (P < 0.05), whereas the relative abundances of *Lactobacillus*, *Veillonella* and *Actinobacillus* were significantly increased in infected suckling piglets relative to uninfected ones (P < 0.05).

**Fig 5:**
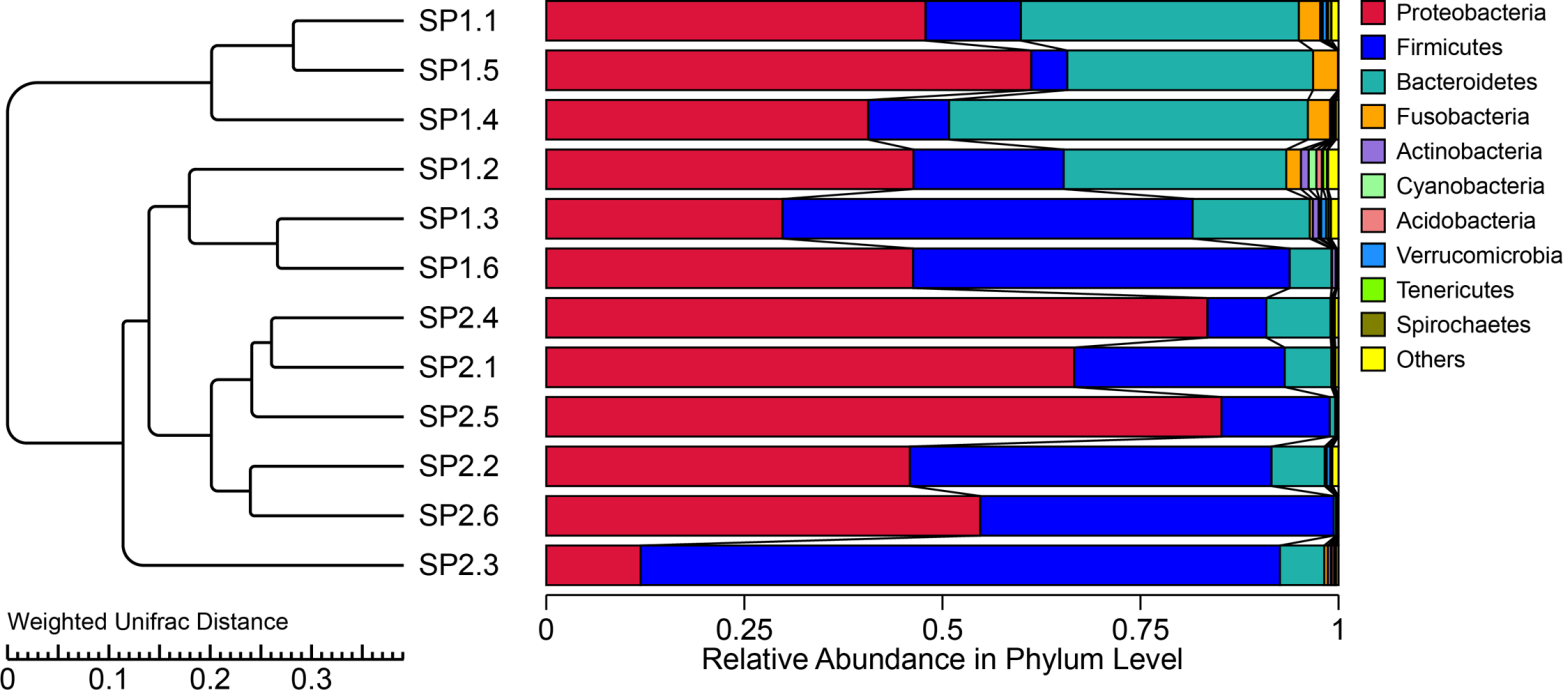
UPGMA phylogenetic tree constructed based on weighted UniFrac distances. The left panel shows the phylogenic tree, and the right panel shows the relative abundance of each sample at the phylum level.

However, the samples also showed variability within each group, and the dilution curve varied among the samples (S1 Fig). To determine the degree of similarity among the samples, a clustering tree of the samples was constructed (Fig 5). All of the samples fell into three major clusters: the samples of the uninfected group fell into two clusters, with the samples of the infected group forming the third cluster. Only one sample from the infected group was located outside of these three clusters.

### Microbial function prediction

**Fig 6:**
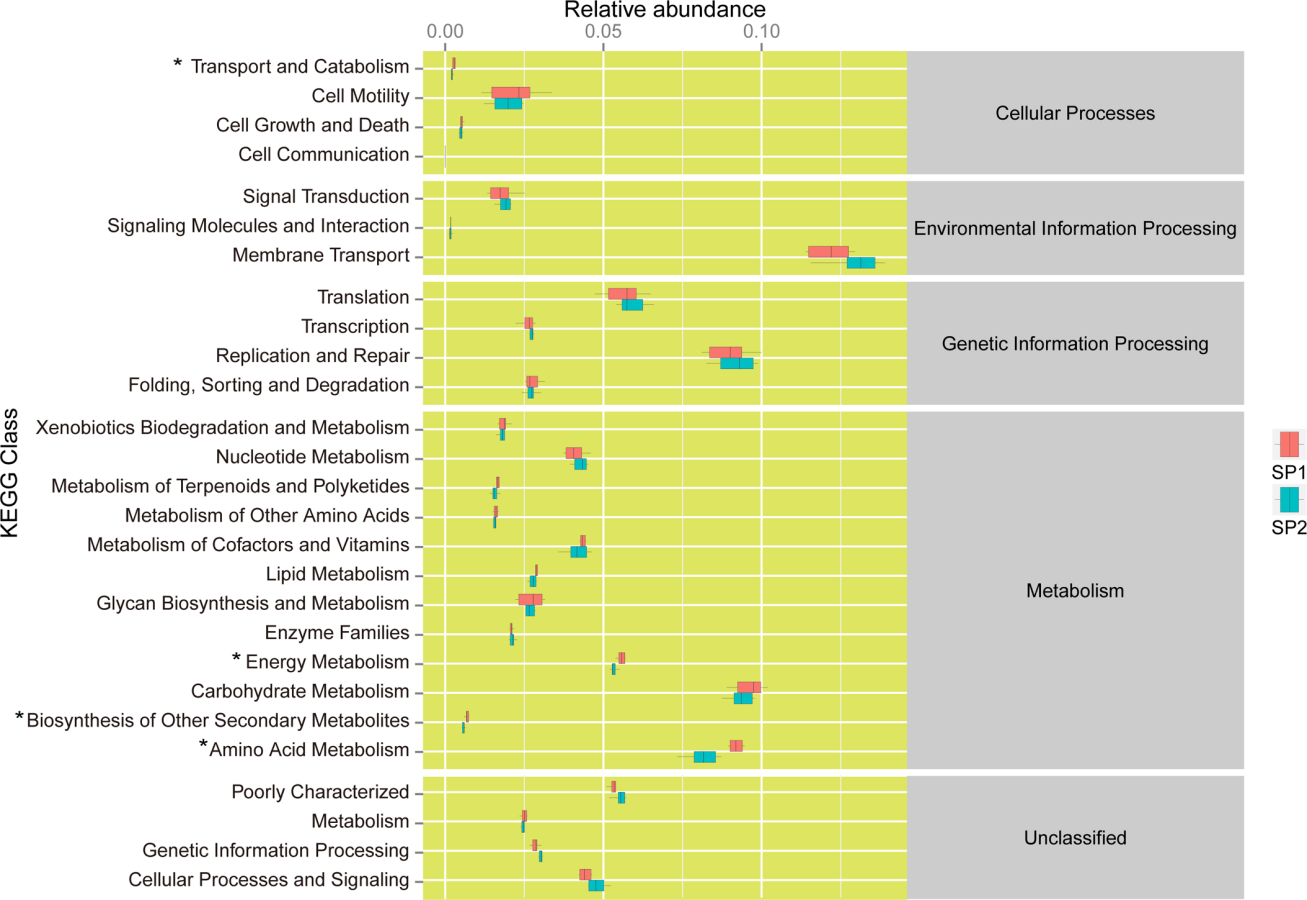
Functional predictions of the small intestinal microbiota of suckling piglets based on the bacterial 16S rRNA gene. * indicates gene categories that are significantly different (P < 0.05) between the control and infected groups.

Microbial function was predicted using PICRUST based on the bacterial 16S rRNA gene. Overall, 27 of 43 level-2 KEGG orthology groups were discovered (Fig 6) in the small intestinal microbiota. The proportion of small intestinal microbiota involved in cellular transport and catabolism, energy metabolism, the biosynthesis of other secondary metabolites and amino acid metabolism was lower in infected suckling piglets than in uninfected suckling piglets (P < 0.05).

## Discussion

Suckling piglets are most susceptible to PEDV at three to seven days of age [4]. Moreover, studies have shown that the first week of suckling is the first phase of intestinal microbial evolution [24,25]. Therefore, seven-day-old suckling piglets were used as subjects in the present study to detect changes in the small intestinal microbiota due to infection with PEDV. It has been shown that age, environment and milk affect the intestinal microbiota [26,27]. To exclude confounding factors and ensure that the study was performed under typical clinical, practical conditions, the sows’ milk was collected, and special enrollment criteria were applied.

Although many researchers have studied the fecal and colonic microbiota of pigs, the small intestinal microbiota has rarely been explored [28–30]. The diversity and abundance of microbiota observed in the present study showed that the small intestinal microbiota of uninfected suckling piglets was simple and dominated by Proteobacteria (49.1%) at the phylum level. The finding is agreement with previous studies that showed that aerobes and facultative anaerobes exhibit obvious superiority in the intestine during the first week of piglet development [11]. The diversity of microbiota confers functional redundancy, which contributes to defense against pathogens [12]. Suckling piglets, which show a simple community structure of intestinal microbiota, have been found to be vulnerable to pathogens [12,31]. The present study found that the composition of the small intestinal microbiota of suckling piglets showed distinct changes from the genus to phylum level under challenge with PEDV, which can be expected to alter the function of the small intestinal flora. Moreover, the significant differences between uninfected piglets and infected piglets in terms of the proportions of microbiota involved in cellular transport and catabolism, energy metabolism, biosynthesis of other secondary metabolites and amino acid metabolism further suggested that the changes in intestinal flora affected their functions. It has been shown that Bacteroidetes and Proteobacteria play important roles in carbohydrate fermentation, the catabolism of polysaccharides and the utilization of amino acids and proteins [11,32,33]. Under the challenge of PEDV infection, the quantity of Bacteroidetes taxa decreased, which might partially explain the lower relative abundance of microbiota associated with energy metabolism, the biosynthesis of other secondary metabolites and amino acid metabolism in the small intestine of infected piglets. In the intestinal microbial ecosystem, many functions of microorganisms, such as carbohydrate fermentation and the catabolism of polysaccharides, are performed in cooperation with bacteria. Therefore, the changes in the relative abundances of *Prevotella*, *Pasteurella*, *Moraxella*, *Lactobacillus*, *Veillonella* and *Actinobacillus* observed under PEDV infection might result in decreases in functions associated with energy metabolism, the biosynthesis of other secondary metabolites and amino acid metabolism in the small intestine of infected piglets.

During the first week of development, suckling piglets have not achieved a stable intestinal microbial ecosystem, which is sensitive to many different external and internal factors. In the complex clinical environment, differences in microorganisms are found within individuals [11,34]. This study similarly found differences in the community composition of the small intestinal microbiota among samples.

Many studies have found that PEDV causes intestinal malabsorption and fatal dehydration due to vomiting and that the timely treatment of diarrhea and vomiting can effectively reduce mortality associated with PED [1]. Microorganisms that play vital roles in material absorption and metabolism have been used to prevent diarrhea [33]. The experimental results indicated that PEDV led to alterations in the community composition of microbes in the small intestine of suckling piglets, including changes in some microbial communities that were associated with metabolism. Therefore, the findings suggested that adjusting the intestinal microbiota might be a promising method for the prevention or treatment of PEDV; however, further studies are needed to confirm this conclusion.

## Conclusion

The small intestinal microbiota of seven-day-old suckling piglets showed poor diversity and was dominated by *Escherichia* (21.4%). Under challenge with PEDV infection, the small intestinal microbiota of suckling piglets showed marked changes, including compositional changes in flora that are associated with cellular transport and catabolism, energy metabolism, the biosynthesis of other secondary metabolites, and amino acid metabolism. The present findings can help identify new preventive measures for treating and controlling PEDV.

## Supporting information

S1 Fig Dilution curve of all of the samples. SP1.1-SP1.6, suckling piglets belonging to the control group; SP2.1-SP2.6, suckling piglets belonging to the infected group.
(TIF)
