# Supplementary figures and images for: Differences in the intestinal microbiota between uninfected piglets and piglets infected with porcine epidemic diarrhea virus
Source: PLoS One. 2018 Feb 15;13(2):e0192992. doi: 10.1371/journal.pone.0192992 (PMC5814011; doi:10.1371/journal.pone.0192992)

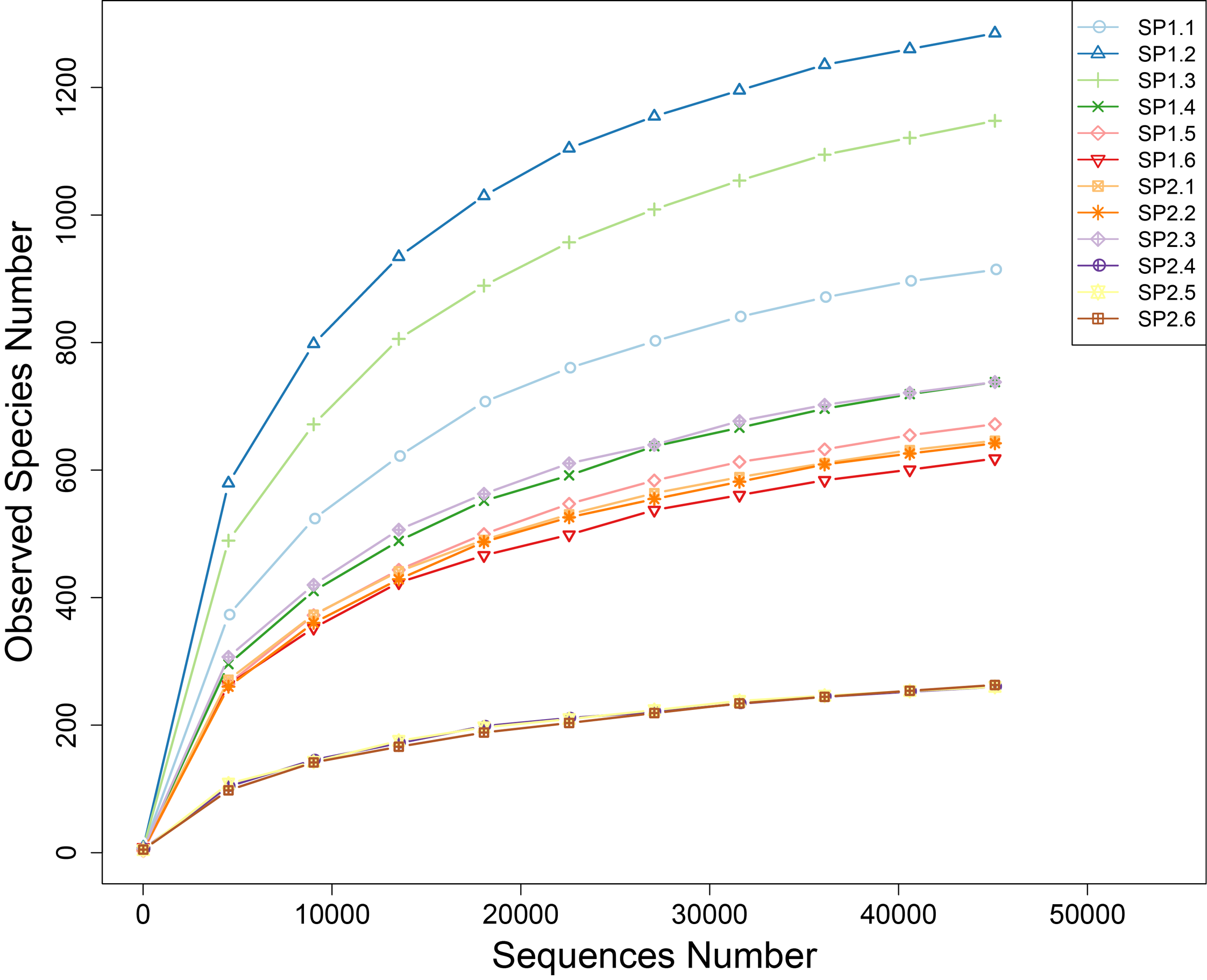

Supplement: S1 Fig — SP1.1-SP1.6, suckling piglets belonging to the control group; SP2.1-SP2.6, suckling piglets belonging to the infected group. (TIF) [file pone.0192992.s001.tif]
